# Rawcopy: Improved copy number analysis with Affymetrix arrays

**DOI:** 10.1038/srep36158

**Published:** 2016-10-31

**Authors:** Markus Mayrhofer, Björn Viklund, Anders Isaksson

**Affiliations:** 1 Science for Life Laboratory, Department of Medical Sciences, Uppsala University, SE-751 85 Uppsala, Sweden; 2 Department of Medical Epidemiology and Biostatistics, Karolinska Institute, Nobels Väg 12A, SE-17177, Stockholm, Sweden

## Abstract

Microarray data is subject to noise and systematic variation that negatively affects the resolution of copy number analysis. We describe Rawcopy, an R package for processing of Affymetrix CytoScan HD, CytoScan 750k and SNP 6.0 microarray raw intensities (CEL files). Noise characteristics of a large number of reference samples are used to estimate *log ratio* and *B-allele frequency* for total and allele-specific copy number analysis. Rawcopy achieves better signal-to-noise ratio and higher proportion of validated alterations than commonly used free and proprietary alternatives. In addition, Rawcopy visualizes each microarray sample for assessment of technical quality, patient identity and genome-wide absolute copy number states. Software and instructions are available at http://rawcopy.org.

DNA copy number alteration is an important mutational process in evolution, population genomics, genetic disorders and cancer development^1^. Gain and loss of gene copies may lead to extreme overexpression, absence of any functional transcript, or modest alterations in gene expression^2^. Genome-wide copy number analysis is commonly performed in hypothesis-generating genomics research, including many recent large-scale cancer studies^3^. It is also growing rapidly in clinical diagnostics as a high-resolution alternative or complement to *in situ* chromosome analysis^4^,^5^.

While recent advances of low-cost sequencing indicate that whole-genome sequencing may eventually become the all-in-one solution for clinical genome analysis, most copy number analysis is currently performed using microarrays^6^. Originally designed for genotyping, several brands of SNP microarrays are now marketed specifically for copy number analysis^7^. Due to their simplicity of operation and relatively manageable data analysis, the use of microarrays for copy number analysis has continued to rise in both cancer and constitutional cytogenetics^8^. It also remains standard practice in cancer genomics studies for which many thousands of samples have been published and made available for data mining by the Cancer Genome Atlas^9^ and at the Gene Expression Omnibus^10^.

DNA microarray signal intensities are subject to noise and systematic variation incurred by factors such as laboratory conditions, reagent quality, non-uniform DNA extraction efficiency along the genome and probe cross-hybridization. This variation limits the resolution and precision by which copy number alterations can be detected and can be quantified using the Median of Absolute Pairwise Differences between adjacent probes (MAPD). Some systematic variation can be removed using patient- or population-matched reference samples processed in an otherwise identical fashion. Systematic variation that affects samples similarly but with different strength, such as GC-content related waviness, can then be further normalized for in individual samples^11^.

Estimating the copy number per cell using extracted DNA from populations of cells has some important limitations. As a fixed amount of DNA is analyzed rather than a fixed number of cells, any multiple of the true set of copy numbers would result in the same observation on the microarray. This is well exemplified by the aneuploidies encountered in cancer genomes, where the total amount of hybridization to the microarray does not reflect the total amount of DNA per cell in the sample. The microarray intensities are median centered to account for variation in total hybridization to the array, with the median intensity corresponding to the median copy number in the genome(s) analyzed. The intensities may also be compared to those of a reference pool of samples or a patient-matched normal sample to account for systematic variation or constitutional copy number variation. Estimation of absolute copy numbers, which has been thoroughly explored in recent years, takes place downstream from basic normalization of raw signal intensities and achieves estimates of the most likely absolute copy numbers given the observations^12^,^13^,^14^.

The normalized intensity per probe relative to the reference is usually log transformed to equalize noise levels over different copy number states, producing the *log ratio*, a measure of DNA abundance along the genome. Saturation effects in microarray hybridization lead to a non-linear relationship between sample DNA abundance and hybridization intensity. Therefore, log ratio is not generally translated into DNA abundance^15^.

Bi-allelic (SNP) probes on the microarray can, in addition to genotyping and heterozygosity mapping, be used for copy number analysis in an allele-specific manner. This results in estimates of the actual number of each parental homologous chromosomal copy per cell^12^,^13^,^14^. Allele-specific signal intensities per SNP probe are usually processed as estimates of the B-allele abundance relative to the total DNA abundance, called the *B-allele frequency* (BAF), ranging from near zero for homozygous A SNPs to near one for homozygous B SNPs.

Downstream analysis of log ratio and BAF includes segmentation (partitioning into segments) of the genome, for which several types of algorithms are in use. Hidden Markov Models use the expected log ratio associated with given copy number states to assign the most likely segment breakpoints given the observations, and are popular in constitutional cytogenetics where the genome can be assumed to be near-diploid and homogeneous. Circular Binary Segmentation (CBS) estimates segment break points without prior assumptions of the amplitude of change incurred by copy number alterations, and is more suitable for cancer genomics where the average ploidy and purity are unknown^16^.

Once segments have been defined, copy number states may be assigned to them based either on deviation from median log ratio, in which case gain and loss are defined relative to the median copy number of the genome, or using more complex analysis of segment log ratio and BAF to estimate the absolute copy numbers per cell. For Affymetrix SNP microarrays, most data analysis is performed using one of the following solutions: Chromosome Analysis Suite (or Genotyping Console for older arrays) is a proprietary solution for Windows systems freely available from Affymetrix. Affymetrix Power Tools is an open source command-line alternative to Chromosome Analysis Suite (ChAS), running on Linux and Mac OS. Nexus Copy Number is commercial software from Biodiscovery Inc., Hawthorne. Other free processing tools for SNP 6.0 have been shown to achieve similar or lower-quality results than Affymetrix Power Tools in previous comparisons^17^,^18^, but not many have been updated to support the current CytoScanHD array. Unfortunately, the general lack of a gold standard in combination with high false-positive rates and profound differences in segmentation strategy between available methods make it difficult to objectively compare performance^19^. We set out to build an open-source solution for processing of Affymetrix SNP 6.0 and CytoScan raw data (CEL files), aiming for better performance than currently available free and proprietary alternatives.

Rawcopy, described here, is a processing tool for Affymetrix CytoScan HD, CytoScan 750k and SNP 6.0 arrays. We demonstrate reduced systematic variation in log ratio and BAF compared to the currently most widely used alternatives, as well as improved prediction accuracy for copy number gain and loss.

Rawcopy is freely available as an installable R package. It is intended to provide the highest quality normalization of log ratio and B-allele frequency, suitable for downstream analysis with a range of tools. It also provides genome segmentation and several visualizations to facilitate assessment of data quality and results. The solutions presented here may also be adopted for processing of other types of microarrays and for sequencing-based copy number analysis.

## Methods

Rawcopy is available as an R package installable under Linux, Mac OS and Windows. Processing time per sample is 10–20 minutes depending on processor speed. The analysis may be run in parallel on multiple processor cores, with each thread requiring less than 8 GB of RAM. Apart from the R package, only sample raw intensity files are required to run the analysis. Reference data are built-in and precompiled from a large number of ethnically diverse samples, with variations also in technical quality (Supplementary Table 1). Users may also use their own reference samples. Rawcopy is available at www.rawcopy.org.

**Figure 1:**
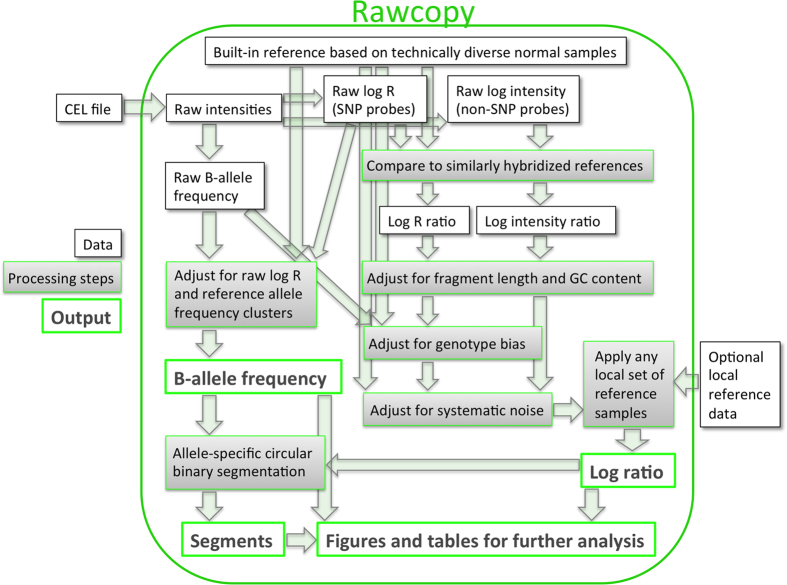
Schematic view of Rawcopy. Grey boxes represent processing steps, white boxes represent data and green borders indicate processed data that form part of the output. The built-in reference data are used in several of the processing steps. The only required input data are the CEL files – standard format of Affymetrix microarray raw intensities. Optional input data are local reference samples. Segmentation is performed using the Parent-Specific CBS method.

The processing of new samples is described in the sections below and is schematically shown in Fig. 1. B-allele frequency for SNP probes is estimated using reference sample genotypes, with normalization for total probe intensity. Total DNA abundance per probe (log ratio) is estimated by comparing total probe intensities to the reference data and normalizing for sample-specific effects such as GC content and fragment length bias. Partitioning of chromosomes into segments of unchanging copy number (segmentation) is performed using the Parent-Specific CBS method^20^. Samples are then further processed to facilitate downstream analysis, including sample identity level matching, estimation of median log ratio and allelic imbalance per gene and genomic segment, and clustering and visualization of the sample set.

Samples are loaded into Rawcopy from raw intensity (CEL) files. Log ratio of SNP probes is based on the Euclidean sum (R) of individual allele A and B mean intensities (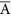 and 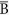. the array contains up to four physical probes for each SNP probe set and allele):


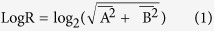


Log ratio of non-SNP probes is based on log_2_ of their raw intensities. Log ratio is composed of both SNP and non-SNP probes, these are merged during the normalization process. BAF is calculated from the raw BAF per SNP probe set:


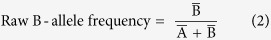


Data processed with Rawcopy are suitable for downstream analysis using a range of tools, including ABSOLUTE^14^, ASCAT^12^, Nexus Copy Number and TAPS^13^.

### Log ratio processing

**Figure 2:**
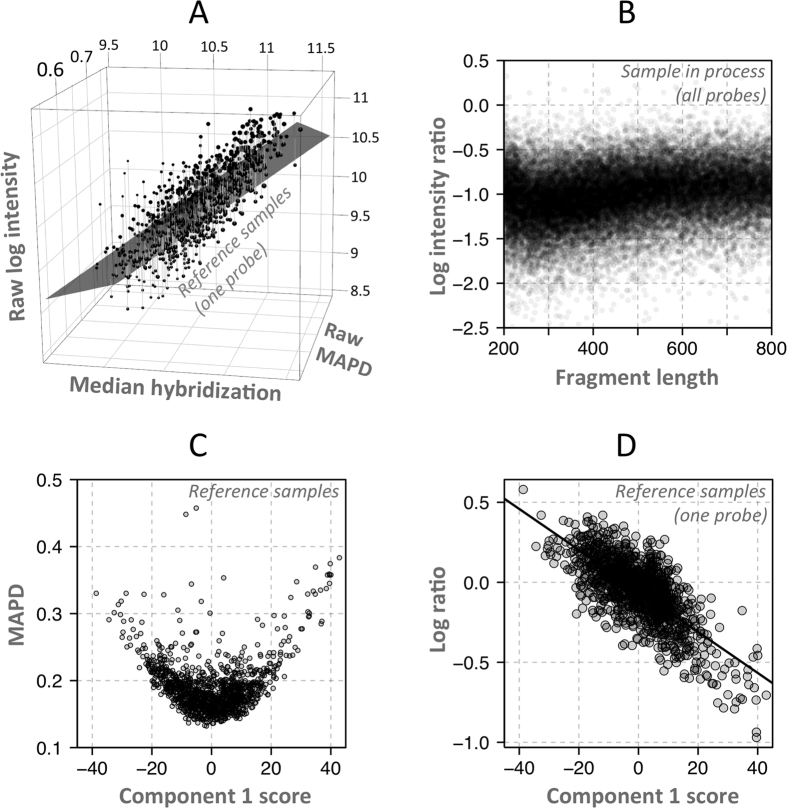
Log ratio processing. (**A**) For each probe, the expected raw intensity is modelled as a linear function of the median hybridization level and raw median absolute pairwise difference (raw MAPD) of the reference samples. Robust linear regression is used to minimize influence of copy number variation, which is assumed to be present in relatively few reference samples for each probe. Only female reference samples were used for the X chromosome and only male samples for the Y chromosome. When processing a new sample, log ratio of each marker is calculated from the difference between the new observation and the reference function value given the median hybridization and raw MAPD of the new sample. (**B**) Example of fragment length bias after the previously described step. Fragment length bias and GC content bias may differ between samples and are therefore only partially corrected for using the reference sample data. Remaining fragment length bias and GC content bias (which may be nonlinear) are corrected for simultaneously by equalizing the median log intensity ratio within percentiles of fragment length and GC content. (**C**) After subjecting reference sample log ratio to multidimensional scaling (MDS), the MDS component scores of reference samples indicated systematic differences between samples, as samples which deviated from the mean along any component were associated with higher MAPD (noise). (**D**) Example of a probe where log ratio correlates with the first MDS component score of each sample.

Log ratio of all probes is calculated using built-in reference data (listed in Supplementary Table 1). For each probe, reference log R (SNPs) or log intensity (non-SNPs) are stored in Rawcopy as a linear function of median log_2_ probe intensity (the amount of hybridization to the microarray) and the raw experimental variation (raw MAPD) of the reference samples, as shown in Fig. 2A. After subtracting the reference value for each probe, given the median hybridization and MAPD of the new sample, log ratio represents logarithmized observations of hybridization relative to the reference level.

Fragment length and GC content bias, which differs between samples (Fig. 2B), are then adjusted for separately in each sample by median-centering the log ratio within percentiles of both fragment length and GC content. Raw BAF is also used to adjust the log ratio of SNPs linearly for correlation between genotype and log ratio in the reference data.

To describe additional systematic variation in the reference material, autosomal log ratio of all reference samples were subjected to multidimensional scaling (MDS), compressing them from one dimension per probe into a few components. The vast majority of variation in the reference material was expected to be noise rather than copy number alterations, and this was also indicated by the data as samples that deviated from the average along any component were associated with more noise (higher MAPD, Fig. 2C). For most probes, the log ratio of reference samples correlated with their component scores (Fig. 2D). This correlation was weaker for each additional component in the MDS (data not shown). Six components were selected as a balance between reducing noise and minimizing data storage in Rawcopy. Linear functions of these six components (for which each sample has a score) are stored in Rawcopy and used to reduce noise for each probe by subtracting the function value, given sample component scores, from the observed log ratio. When processing a new sample, the score giving the lowest MAPD is determined and used for each of the six components.

If a local set of reference samples is available, a local reference file can be built and used to further reduce noise and waviness. This is achieved by subtracting the median local reference log ratio from that of the query sample, for each probe.

### B-allele frequency estimation

**Figure 3:**
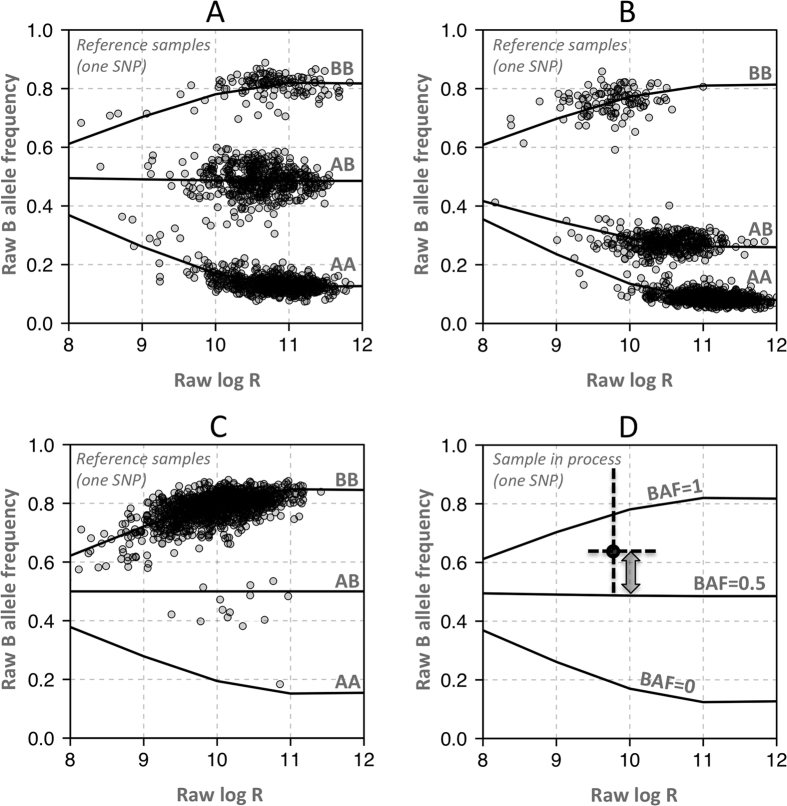
BAF estimation. (**A**) Reference samples were used to express raw B-allele frequency as functions of raw log R given genotypes AA, AB and BB, for all SNPs. To reduce extrapolation problems, a characteristic function was designed based on the general behavior of the data, with parameters that could be fitted for individual SNPs. SNPs with good separation of clusters and with each cluster populated by multiple samples were considered high-quality. (**B**) Robustness against hybridization bias between genotypes was confirmed by visually inspecting a large number of SNPs. (**C**) SNPs with poor separation of clusters and/or difficulty of defining cluster centers were considered low-quality. For them, the mirror image around 0.5 of the most well-defined homozygous cluster was used instead of the poorly represented homozygous genotype, and a straight line at 0.5 was used for heterozygous SNPs. (**D**) When processing new samples, BAF is calculated using the stored function parameters for each SNP and genotype cluster, and the raw BAF and log R of the new sample. In this example, raw BAF (black dot) appears above the function line for the AB genotype. BAF is therefore calculated linearly from the observation relative to the AB (BAF = 0.5) and BB (BAF = 1) function lines, yielding a value near 0.75.

Due to background hybridization and the possibility of unequal specific and non-specific hybridization of the two alleles, the raw BAF defined in Formula 2 cannot be assumed to accurately represent the true BAF. In Rawcopy, the raw BAF associated with each normal diploid genotype (AA, AB and BB) in the reference material is stored in Rawcopy as a function of the “log R” defined in Formula 1. Examples of SNPs with well-defined genotype clusters are shown in Fig. 3A,B.

A subset of SNPs (35% of CytoScan, 44% of SNP 6.0) displayed either poorly separated clusters or no variation in genotype among the reference samples. The reference data for those SNPs are therefore considered low-quality. An example of such a SNP is shown in Fig. 3C. The criteria used for high-quality SNPs was three clusters and a total cluster sum of squares relative to the number of reference samples of at least 0.018. Inclusion of low-quality SNP data is optional in Rawcopy. Heterozygosity rates for SNPs associated with high- and low-quality reference data are presented in Supplementary Fig. 1. When processing new samples, Rawcopy estimates the BAF of each SNP as shown in Fig. 3D, given the observed “log R” and raw BAF.

### Genome segmentation

The segmentation step available in Rawcopy uses the PSCBS package^20^. Once segment break points have been determined, segments are annotated with median log ratio, number of probes, genes and cytoband. Allelic imbalance for genomic segments is quantified in the same way as in TAPS^13^. Segment tables are written to tab-separated text files for browsing and further analysis.

### Data visualization

**Figure 4:**
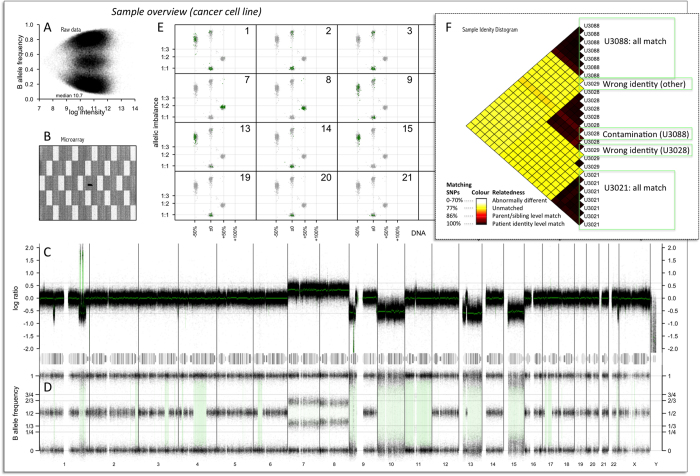
Rawcopy visualization per sample (**A–E**) and data set (**F**). (**A**) A scatter plot of raw log intensity versus raw B allele frequency shows the overall hybridization and genotype cluster distribution of the sample. (**B**) Raw signal intensity throughout the microarray may indicate uneven hybridization and array quality issues. (**C**) Log ratio indicates alterations in total copy number along the genome. Segments are marked in green and small amplifications and deletions are highlighted for visibility. (**D**) B-allele frequency indicates the genotype of each SNP probe. Most copy number alterations lead to allelic imbalance indicated by absence of the middle band of AB genotype SNPs. Allelic imbalance and homozygous segments are highlighted for visibility. (**E**) Scatter plots of segmented DNA abundance estimates and allelic imbalance, with individual chromosomes highlighted relative to the rest of the genome, indicate absolute copy number per cell and any heterogeneity in the cell population. (**F**) Genotype data from all samples in the batch are cross-matched and patient identity level dissimilarity scores are computed. This example shows multiple cancer cell lines from four tumors. Two examples of erroneous sample identity are indicated in the distogram; one U3029 sample matches U3028 instead and one U3029 sample matches none of the other samples. In addition one U3028 sample shows some cell or DNA contamination from U3088.

After processing of log ratio, BAF and segmentation, whole-genome and chromosome-wise figures are plotted for each sample. These allow the user to assess technical quality of individual samples such as total hybridization level and quality of the physical array, and get an extensive overview of chromosomal alterations as shown in Fig. 4.

To visualize the copy number throughout the genome, scatter plots of total copy number and allelic imbalance are shown throughout each chromosome relative to the rest of the genome (Fig. 4E). This is equivalent to a previously published solution^13^,^15^ and can be used to indicate the absolute number of copies involved in copy number alterations, mosaicism, and the ploidy and purity of cancer samples. In the scatter plots, the median log ratio of each segment (about 1 Mb long) is transformed into estimates of DNA abundance relative to the median of the current sample. Cancer cell line samples^21^ were used to set the expected log ratio given 50% loss (log ratio: −0.6), 50% gain (log ratio: 0.35) and 100% gain (log ratio: 0.6) of DNA abundance relative to the median of the genome. (Individual samples may deviate slightly from this model for technical reasons.) Allelic imbalance is measured for each segment by first clustering abs(BAF-0.5) on two means, representing the separation of heterozygous and homozygous SNPs (BAF_het_ and BAF_hom_), then quantifying the separation of BAF_het_ relative to BAF_hom_:


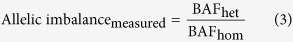


Assuming heterozygous SNPs exist and are separated into two bands due to imbalanced copy number, allelic imbalance represents estimates of the absolute difference in the copy number of each parental homologue (H) relative to their sum (the total copy number):


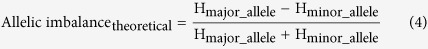


Due to noise in BAF estimates, segments where the copy number is balanced or homozygous result in measured allelic imbalances just above 0 or below 1, respectively (Fig. 4E). The scatter plots are annotated with the expected allelic imbalance given a 1:1, 1:2 and 1:3 ratio of homologous copies.

The pairwise genotype dissimilarities of all samples processed together are plotted as shown in Fig. 4F. BAF is discretized into B-allele presence (1 if BAF ≥ 0.2, else 0), reducing the effect of systematic and copy number variation while largely retaining genotype information on sample identity level. Pairwise dissimilarities (sum of differences in B-allele presence) between samples are then visualized in a distogram^22^, using a color gradient based on observed dissimilarities between related and unrelated members of HapMap CEU. In addition to validating sample identities relative to one another, the sample identity distogram may indicate cell or DNA contamination.

## Results and Discussion

Two large sets of publicly available samples were acquired from the Gene Expression Omnibus (GEO) for systematic benchmarking of performance relative to some of the most commonly used free and proprietary processing tools. For the Affymetrix SNP 6.0 platform, a set of 947 cancer cell lines^23^ published by the Broad Institute, Massachusetts (GEO accession number: GSE36138) was analyzed using Affymetrix Power Tools, Nexus Copy Number and Rawcopy. For the Affymetrix CytoScan HD platform, a set of 231 hepatocellular carcinomas^24^ published by the Gachon University of Incheon, South Korea (GEO accession number: GSE54504) was analyzed using Affymetrix Power Tools, Nexus Copy Number, ChAS and Rawcopy. In addition, this set of samples was analyzed with Rawcopy using the included matched normal samples as local reference data to further reduce noise.

### Reduced log ratio noise relative to true signal

The most commonly used metric of technical quality in microarray copy number analysis is MAPD which estimates the amplitude of log ratio noise in a way that is largely unaffected by copy number alterations. As the majority of adjacent measurements of log ratio should be the same, the median of their absolute pairwise differences are frequently relied upon to compare technical quality across samples with different distributions of copy number states. However as some methods employ normalization steps that alter the distribution of the data, such as quantile normalization, the MAPD may not be comparable across different processing tools. In cancer samples with large copy number alterations, MAPD may be adjusted based on the observed effects of copy number alteration on log ratio. We defined the signal-adjusted pairwise difference (SAPD) as the MAPD divided by the effect Δ of copy number alteration on log ratio with the current processing tool, relative to the average effect 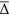 over a set of different processing tools (given the same sample and copy number alteration):


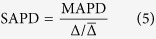


**Figure 5:**
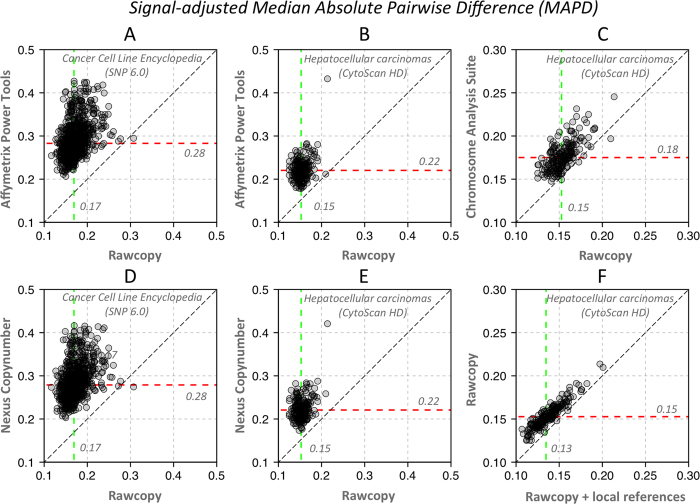
Noise estimates for Rawcopy and other processing tools. Signal-adjusted Pairwise Difference (SAPD) of evaluation samples with Rawcopy and commonly used alternatives (lower values are better). Colored dashed lines indicate median SAPD. Rawcopy achieved significantly better SAPD than each of the alternatives for both the CytoScan HD and SNP 6.0 evaluation sets (p < 2.2_*_10^−16^, paired Student’s T-tests). (**A**) Affymetrix Power Tools and SNP 6.0. (**B**) Affymetrix Power Tools and CytoScan HD. (**C**) ChAS and CytoScan HD. (**D**) Nexus Copy Number and SNP 6.0. (**E**) Nexus Copy Number and CytoScan HD. **F** Use of local reference samples (patient-matched normals used as a local reference data set) further reduced Rawcopy SAPD.

MAPD and SAPD were calculated for each sample and each processing tool in the evaluation. For each sample in the evaluation data, the two autosomes with the highest and lowest median log ratio was selected for calculating Δ with all processing tools. Samples with little or no evidence of copy number alteration were removed (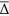 less than 0.2). SAPD of the evaluation samples are shown for Rawcopy and the commonly used current processing tools in Fig. 5.

### Improved estimates of B allele frequency

**Figure 6:**
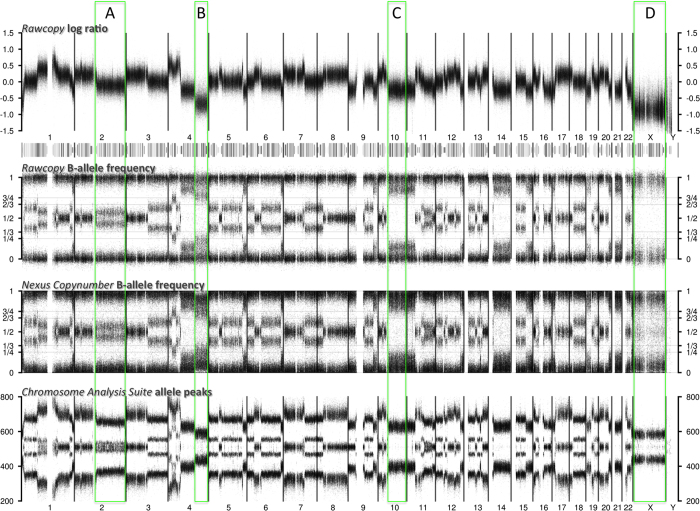
Representative example (GEO:GSM131277_H061350T) of log ratio, BAF and Allele Peaks from Rawcopy, Nexus Copy Number and ChAS. (**A**) In the BAF tracks the middle band of heterozygous SNPs has split, indicating some allelic imbalance. ChAS allele peaks do not show this clearly throughout the chromosome arm, oscillating instead between a balanced (one middle band of SNPs) and unbalanced profile. (**B**) At low total copy number, Nexus BAF shows some bias as homozygous SNPs, with expected BAF near 0 or 1, have shifted towards 0.5. With Rawcopy, homozygous SNPs remain steadily near 0 or 1, indicating that such bias is successfully normalized for. ChAS allele peaks do not clearly indicate separation between homozygous (outer bands) and heterozygous (inner bands) SNPs. (**C**) Rawcopy BAF shows distinction between homozygous and heterozygous SNPs, indicating that the abundance of the minor allele is relatively low but not zero. (**D**) With Rawcopy, all SNPs of the homozygous X chromosome (of this male sample) appear near 0 or 1. With Nexus, BAF appears closer towards the center as the limited amount of DNA leads to less separation of A and B clusters than expected given normal copy number. In addition, with Rawcopy, SNPs with low hybridization are less prone to appear heterozygous than with Nexus or ChAS (where seemingly heterozygous SNPs appear despite a homozygous X chromosome).

Rawcopy and Nexus Copy Number both provide estimates of the B allele frequency for each SNP, but Nexus Copy Number truncates the data at zero and one. ChAS provides Allele Difference (sometimes called Allele Peaks) representing the difference between log_2_(A) and log_2_(B). These different approaches result in similarly useful but somewhat different allelic data that is shown in Fig. 6. Rawcopy and Nexus achieve better separation and stability of SNPs with near-equal abundance of the A and B allele compared to ChAS (6A). Rawcopy also achieves the best separation of homozygous and near-homozygous SNPs (6B-C). Rawcopy BAF is less skewed by total DNA abundance than Nexus BAF (6B,D). To obtain a quantitative measure of the quality of the BAF normalization (Rawcopy and Nexus) the standard deviation of heterozygous SNPs (BAF 0.75 to 0.25) for each HapMap sample was measured and found to be 0.059 on average for Nexus and significantly lower for Rawcopy with 0.048 on average (p < 2.2*10^−16^). Rawcopy uses a subset of high-quality SNP probes while Nexus uses all SNP probes. To control for this difference we measured the standard deviation of the BAF normalized with Nexus for the high-quality heterozygous SNPs used by Rawcopy and found it to still be significantly higher than for Rawcopy (0.052 on average p = 5*10^−6^). A similar comparison with variation in allelic signals provided by ChAS could not be performed since ChAS only provides Allele Difference.

### Prediction accuracy for copy number alterations

**Figure 7:**
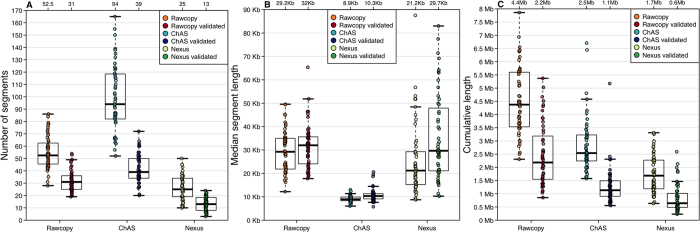
Characteristics of altered segments detected in CytoscanHD data from 52 HapMap trios using Rawcopy, ChAS and Nexus. (**A**) Number of altered segments detected in the child and validated in parent samples. Median values are printed above each set of values. (**B**) Median segment length, for each child, of detected and validated alterations. (**C**) Cumulative length of detected and validated alterations in each child.

**Figure 8:**
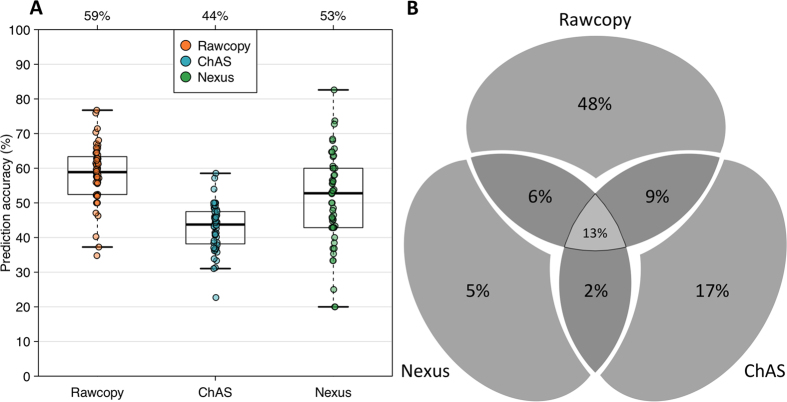
Characteristics of validated alterations in 52 HapMap trios. (**A**) Distribution of prediction accuracy in 52 HapMap trios for Rawcopy, ChAS and Nexus. (**B**) The median percent overlap of validated altered sequences in the 52 HapMap trios detected with Rawcopy, ChAS and Nexus is shown in a Venn diagram.

The ability to identify alterations identical by decent was investigated for Rawcopy, ChAS and Nexus Copy Number, using 52 HapMap trios analyzed on Affymetrix current generation of high-density SNP arrays (CytoScanHD). Data normalized with Rawcopy or Nexus Copy Number were segmented using the same method (rank CBS in Nexus) to avoid any differences introduced by segmentation settings. Segment median log-ratio thresholds were >0.2 for gains and <−0.3 for deletions. ChAS was run with its default HMM segmentation. For each method, alterations detected in children were considered validated if also detected with at least 90% overlap in one parent. Total number of altered segments and median and cumulative segment lengths in the trios are shown for all three methods in Fig. 7A–C. ChAS identified and the most numerous but relatively short alterations, while Rawcopy detected the longest cumulative alteration length per sample (median 4 Mb compared to 2.5 Mb for ChAS, p = 7.7*10^−11^). Nexus produced the smallest median of both total number of alterations and cumulative length. Rawcopy showed the highest prediction accuracy (calculated as previously by Nutsua *et al*.^18^) as shown in Fig. 8A, Median prediction accuracy for Rawcopy was 59%, significantly higher than ChAS (44%, p < 2.2*10^−16^) and Nexus (53%, p = 0.0010). The proportion of overlap between validated alterations detected with the different methods was calculated for each trio and their medians are shown in Fig. 8B. A median of 48% of the validated alterations were uniquely detected by Rawcopy, consistent with Rawcopy’s larger cumulative length of detected alterations (7C).

### Significance

Rawcopy makes copy number analysis easy to set up, as only installation of the R-package is required to start processing CEL files. The large built-in reference data lead to better quality of the copy number data, i.e. reduced noise relative to signal, better prediction accuracy and more accurate BAF compared to the most widely used free and proprietary alternatives. A noise level threshold such as MAPD is the most commonly used quality metric for SNP microarrays, but differences in the signal distribution achieved by different processing tools preclude direct comparison of MAPD between them. MAPD was corrected for such differences, allowing us to compare noise between processing tools. Use of SAPD over MAPD is not suggested when processing new samples in general as it is intended for comparing tools, not samples. All new samples cannot be expected to harbor copy number alterations of sufficient length and amplitude to make signal-to-noise assessment practical.

Figures generated by Rawcopy allow immediate assessment of individual sample quality and copy number profile, and technical issues such as DNA quality and microarray fabrication errors can be identified on individual arrays. The sample identity distogram can indicate mislabeled samples and reveal DNA or cell contamination.

Rawcopy is suitable for copy number and heterozygosity analysis of both tumour and constitutional DNA. Absolute allele-specific copy numbers may be estimated using scatter plots of median log ratio versus allelic imbalance for genomic segments, even for cancer samples with extensive aneuploidy, low purity or subclonal heterogeneity^13^. Copy number analysis of DNA extractions from populations of cells is ambiguous in nature as there may be more than one set of absolute copy numbers per cell that would explain the composition of a DNA sample. With Rawcopy, we have built a tool capable of revealing rich information about the copy number profile, but without applying any automatic classification or interpretation (such as purity and ploidy for cancer samples) that could fail for samples with an unforeseen chromosomal setup. Arguably such interpretations should be done taking into account the specific disease and its frequency of specific karyotypes and genome doublings, as done in ABSOLUTE^14^. However, even then many samples cannot be correctly resolved as alternative solutions may be near-equally plausible. If the interpretation impacts diagnosis or other clinical decisions, cell-based chromosome or ploidy analysis may be motivated as a complement to the microarray. If specific estimates of genome-wide of absolute allele-specific copy numbers (i.e. numeric data for further analysis) are required, the output generated by Rawcopy is suitable for downstream analysis tools such as ABSOLUTE, ASCAT or TAPS. The reduction of systematic BAF bias and reduced BAF variation achieved in Rawcopy also makes BAF a more accurate representation of true allele ratio and more likely to fit theoretical models of cell fractions with certain copy number states.

## Conclusion

Rawcopy is a freely available R package that provides improved normalization of Affymetrix SNP arrays for copy number analysis. It achieves improved signal-to-noise ratio and prediction accuracy compared to commonly used alternatives. Rawcopy also facilitates interpretation of complex and heterogeneous copy number profiles through visualization of log ratio and allelic imbalance, and the output is compatible with several alternatives for downstream analysis. Included in the package is also a powerful feature for plotting of SNP genotype dissimilarities between samples in a batch, which may be indicative of DNA contamination or mislabeled sample identities. Using this feature helps ensure that there are no apparent patient identity level errors in the data set.

### Availability and requirements

Project name: Rawcopy

Project home page: http://rawcopy.org

Operating systems: Linux, OSX and Windows

Programming language: R

Other requirements: Minimum 8GB of RAM

License: GNU General Public License

Any restrictions to use by non-academics: No

### Availability of data and materials

Rawcopy is free software and may be redistributed and/or modified under the terms of the GNU General Public License as published by the Free Software Foundation; version 2. Installation, execution and access are described at http://rawcopy.org. The set of 947 cancer cell lines is available at GEO with accession number GSE36138. The set of 231 hepatocellular carcinomas is available at GEO with accession number GSE54504. The individual SNP 6.0 reference samples (BRCA, COAD, GBM and LUAD non-cancer samples were used as references) as well as samples used as examples can be obtained from TCGA upon request (https://tcga-data.nci.nih.gov/tcga/). Individual Swedish clinical CytoScan HD reference samples are not publically available. Individual HapMap CytoScan HD/750k reference samples can be obtained from Affymetrix Inc. upon request.

### Ethics

The research use of the clinical samples collected in Uppsala, Sweden, was approved by the Regional Ethical Review Board in Uppsala (2010/236). Informed consent was obtained from all patients and all experiments were performed in accordance with relevant guidelines and regulations.

## Additional Information

**How to cite this article**: Mayrhofer, M. *et al*. Rawcopy: Improved copy number analysis with Affymetrix arrays. *Sci. Rep.* **6**, 36158; doi: 10.1038/srep36158 (2016).

**Publisher’s note:** Springer Nature remains neutral with regard to jurisdictional claims in published maps and institutional affiliations.

## Supplementary Material

Supplementary Information
