# Supplementary material for: Rawcopy: Improved copy number analysis with Affymetrix arrays
Source: Sci Rep. 2016 Oct 31;6:36158. doi: 10.1038/srep36158 (PMC5086940; doi:10.1038/srep36158)
Supplement: Supplementary Information [file srep36158-s1.doc]

# Rawcopy: Improved copy number analysis with Affymetrix arrays

Markus Mayrhofer, Björn Viklund and Anders Isaksson

# Supplementary Data

Supplementary Table 1

Supplementary Figure 1

*Supplementary Table 1. Samples used for Rawcopy internal reference data.*

| **Array type** | **Source** | **Sample type** | **Number** |
| --- | --- | --- | --- |
| CytoScan HD | Uppsala Array Platform | Constitutional DNA Sweden* | 854 |
| CytoScan HD | Affymetrix | HapMap CEU | 191 |
| CytoScan HD | Affymetrix | HapMap CHB | 48 |
| CytoScan HD | Affymetrix | HapMap JPT | 43 |
| CytoScan HD | Affymetrix | HapMap YRI | 97 |
| SNP 6.0 | The Cancer Genome Atlas | BRCA, COAD, GBM and LUAD (normal DNA only) | 1642 |

**Clinical samples, research use was approved by the Regional Ethical Review Board of Uppsala (2010/236).*


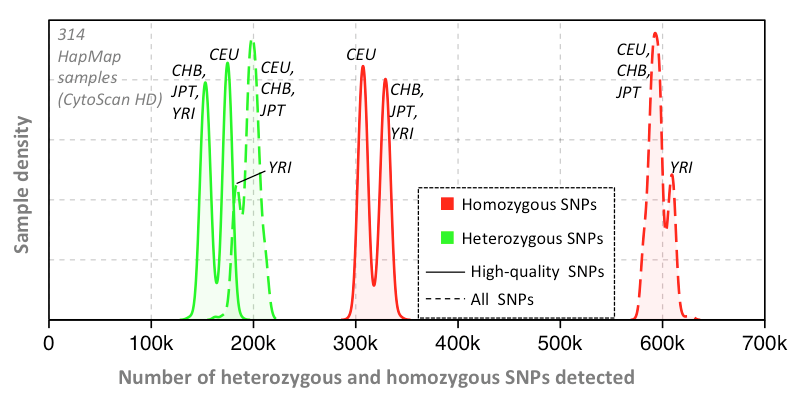


***Supplementary Figure 1****: Density plots of the number of heterozygous (green, informative for allelic copy number analysis) and homozygous (red, uninformative) SNPs, using all SNPs (dashed lines) or high-quality SNPs only (solid lines). With the CytoScan HD SNP set, HapMap populations separate slightly with CEU (Utah residents with ancestry from northern and western Europe) at higher and YRI (Yoruba in Ibadan, Nigeria) at lower heterozygosity rates than CHB (Han Chinese in Beijing, China) and JPT (Japanese in Tokyo, Japan). With Rawcopy, omitting low-quality SNPs improves the heterozygosity rate from about 25% to about 35% at the cost of losing 10%(CEU)-25%(CHB, JPT) of heterozygous SNPs.*
